# Laser Fabrication of 3D Gelatin Scaffolds for the Generation of Bioartificial Tissues

**DOI:** 10.3390/ma4010288

**Published:** 2011-01-19

**Authors:** Aleksandr Ovsianikov, Andrea Deiwick, Sandra Van Vlierberghe, Michael Pflaum, Mathias Wilhelmi, Peter Dubruel, Boris Chichkov

**Affiliations:** 1 Department of Nanotechnology, Laser Zentrum Hannover e.V., Hollerithallee 8, D-30419 Hannover, Germany; E-Mails: a.deiwick@lzh.de (A.D.); b.chichkov@lzh.de (B.C.); 2 Polymer Chemistry & Biomaterials Research Group, University of Ghent, Krijgslaan 281, Building S4-Bis, 9000 Ghent, Belgium; E-Mails: sandra.vanvlierberghe@ugent.be (S.V.); peter.dubruel@ugent.be (P.D.); 3 Department of Cardiac-, Thoraic-, Transplantation-, and Vascular Surgery, Hannover Medical School, Carl-Neuberg-Strasse 1, D-30625 Hannover, Germany; E-Mails: pflaum.michael@mh-hannover.de (M.P.); wilhelmi.mathias@mh-hannover.de (M.W.)

**Keywords:** scaffolds, computer-aided design, laser fabrication, two-photon polymerization, gelatin, tissue engineering, biodegradation, stem cells, adipose tissue

## Abstract

In the present work, the two-photon polymerization (2PP) technique was applied to develop precisely defined biodegradable 3D tissue engineering scaffolds. The scaffolds were fabricated via photopolymerization of gelatin modified with methacrylamide moieties. The results indicate that the gelatin derivative (GelMod) preserves its enzymatic degradation capability after photopolymerization. In addition, the developed scaffolds using 2PP support primary adipose-derived stem cell (ASC) adhesion, proliferation and differentiation into the anticipated lineage.

## 1. Introduction

Scaffold fabrication is one the most rapidly evolving technological aspects related to tissue engineering. A large variety of materials including natural, synthetic, and combinations thereof have already been applied as starting materials during the last two decades [1,2,3]. Ideally, scaffolds should mimic the natural environment of the targeted tissue. Although the applied material determines the cell-interactive properties to a large extent, recent findings indicate that the scaffold geometry plays a pivotal role in its ability to mimic the natural cellular environment [4,5,6,7,8]. Using conventional approaches including freeze-drying, phase separation, solvent casting and electrospinning, pore connectivity and average pore size can already be controlled. However, the final scaffold architecture cannot be fine-tuned using conventional techniques. Recently, a series of technologies was elaborated enabling production of scaffolds possessing a well-defined pore shape, size, distribution and interconnectivity starting from a provided design [9,10,11]. The most frequently applied techniques include 3D fiber deposition, fused deposition modeling, selective laser sintering and stereolithography. Using these additive manufacturing technologies, the fabrication of computer-aided design (CAD) scaffolds with pore size ranging from ten to hundreds of micrometers and high spatial resolutions becomes possible. The properties of the produced scaffolds depend to a great extent on the applied method and the selected starting material.

We have previously demonstrated the feasibility to apply two-photon polymerization (2PP) to develop 3D scaffolds starting from conventional photosensitive materials [12] and acrylated PEG [13]. Two more recent reports described the development of 3D structures from potentially biodegradable materials by 2PP-fabrication [14,15]. This method is related to stereolithography, since it uses materials photopolymerizable by UV radiation. In case of 2PP, multi-photon absorption of laser radiation by the material is used to trigger highly localized photopolymerization reaction. The most important advantages of 2PP include its superior resolution and the possibility to produce complex 3D structures directly, without the necessity for layer-by-layer deposition of the material.

Engineering adipose tissue substitutes which promote tissue regeneration is a key issue in plastic and reconstructive surgery [16]. In order to develop a functional substitute, the 3D tissue architecture has to be mimicked to provide sufficient integration after implantation. The necessity of 3D cell culture for adipose tissue engineering has already been emphasized by Fischbach *et al*. [17]. They reported on the development of fat pads starting from preadipocytes seeded onto a 3D polyglycolic acid-based fiber mesh. The study demonstrated that in contrast to conventional 2D monolayer methods, a 3D tissue culture setup enabled the production of uniform tissue histologically comparable to native fat. The results were attributed to the created 3D environment facilitating cell-cell and cell-ECM (Extracellular Matrix) interactions. Few further reports evaluated the possibility to use scaffolds possessing a random pore distribution for adipose tissue engineering. The reported scaffold materials include biodegradable polymer sponges [18,19], collagen [20], hyaluronic acid [21] and PEG [22]. A majority of the current autologous transplants experience failure due to mechanical damage and/or insufficient vascularization of the *de novo* formed adipose tissue. An attractive solution is the application of 3D scaffolds enabling appropriate cell adhesion, migration and subsequent organization in order to develop adipose tissue transplants. In addition, the developed scaffolds should provide a structural support protecting cells and tissue from mechanical damage induced by the transplantation procedure. Cho *et al.* already described the feasibility to apply scaffolds in order to protect cells from mechanical damage. In their study, functional adipose tissue was generated *in vivo* [23]. It should, however, be pointed out that the biodegradable, dome-shaped constructs did not function as cell carriers as such, but were instead combined with cell-fibrin mixtures via injection after scaffold implantation.

Numerous reports have already described the application of 3D scaffolds for adipose tissue formation. However, since most of these studies applied scaffolds with a random pore distribution, little is known about the influence of the scaffold architecture on the subsequent adipose tissue formation. In the present work, we report on the development of 3D scaffolds using 2PP starting from photosensitive gelatin. Both the high resolution and the reproducibility of 2PP enable the fabrication of a series of identical structures. As a result, systematic studies on 3D adipose tissue formation can be performed.

The photopolymerizable material applied in the present work was obtained by chemically modifying gelatin with methacrylamide moieties. The resulting GelMod therefore combines the biomimetic properties of natural gelatin with the advantages of synthetic materials (*i.e*., tunable mechanical properties) [24]. A gelatin-based aqueous solution is photopolymerized in the presence of the photoinitiator Irgacure 2959. In order to assess the suitability of the developed 2PP scaffolds for tissue engineering applications, they are seeded with primary human adipose-derived stem cells (ASC).

## 2. Results and Discussion

### 2.1. Methacrylamide-Modified Gelatin (GelMod) and Its *in vitro* Degradation Behavior

**Figure 1:**
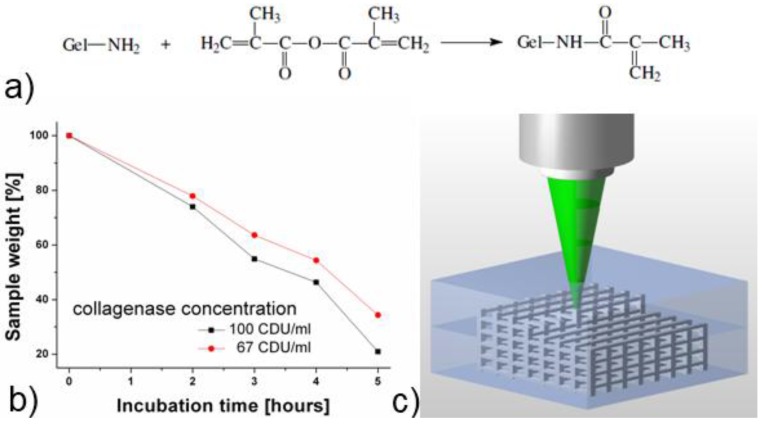
(**a**) Overview of the synthesis of methacrylamide- modified gelatin (GelMod); (**b**) Degradation behavior of photopolymerized gelatin in collagenase solutions; and (**c**) Schematic principle of the 2PP technique.

The GelMod synthesis is schematically depicted in **Figure 1****a**. The modification degree (*i.e.*, degree of substitution, DS), is defined as the ratio of the amount of incorporated methacrylamide functionalities to the amount of free amine groups available for modification (*i.e*., lysine and hydroxylysine present in the gelatin backbone). The DS can be finetuned by varying the amount of methacrylic anhydride added during the synthesis [25]. The modification degree determines to a large extent the mechanical properties of the final polymer scaffold. In the present work, GelMod with a DS of 65% was selected.

In order to evaluate whether GelMod preserves its enzymatic degradation ability, photopolymerized material pellets were incubated in collagenase solutions possessing different concentrations. The degradation behavior was quantified by monitoring the sample mass as a function of the incubation time. As anticipated, increasing the collagenase concentration accelerated the degradation process of photopolymerized GelMod (**Figure 1****b**). Immersing the samples for five hours into a solution corresponding to 67 CDU/mL (collagenase digestion units), resulted in a sample weight decrease to 35% of the initial value. After incubation in a solution corresponding to 100 CDU/mL, only 20% of the initial sample weight remained after five hours. Based on the observed degradation behavior and on the sample appearance, we anticipate that bulk degradation occurred. This degradation mechanism was already observed before for porous gelatin-based scaffolds prepared by a cryogenic treatment followed by lyophilization [25]. Since the enzymatic degradation capability of methacrylamide-modified gelatin is preserved after its photopolymerization, cell-secreted enzyme-mediated degradation of the 2PP scaffolds should be feasible [26]. Therefore, scaffolds composed of GelMod are potentially cell-responsive since they offer cells the possibility to spatially and temporally control their surrounding environment. This dynamic dialogue has already been described before as one of the key features to realize tissue regeneration [27].

### 2.2. Scaffold Fabrication Using Two-Photon Polymerization (2PP)

**Figure 2:**
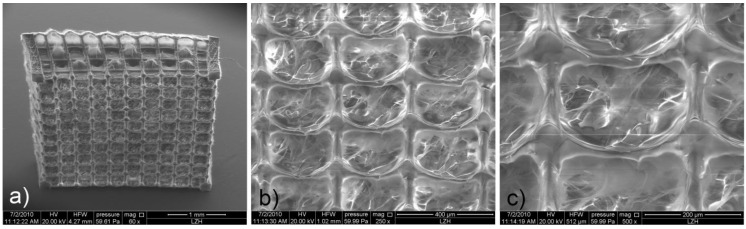
SEM images of the 2PP-produced gelatin scaffolds: (**a**) an overview image; (**b,c**) magnified images showing that the scaffold pores are filled with a fibrous mesh.

The scaffolds were produced by direct laser writing using two-photon polymerization (2PP) with a femtosecond laser emitting at around 515 nm. In addition to its biocompatibility, the added photoinitiator (*i.e*., Irgacure 2959) was selected since its absorption spectrum matches the half-wavelength of the applied laser radiation. As a result, Irgacure 2959 is well suited to enable 2PP processing. The tightly focused femtosecond laser beam interacts with the material via a two-photon absorption process. The subsequent photopolymerization reaction occurring in the focus of the laser beam renders a highly localized volume of the GelMod insoluble. By moving the focal spot throughout the material, photopolymerized 3D patterns are “recorded” (**Figure 1****c**) enabling the fabrication of complex structures with high spatial resolution. In a final step, the unpolymerized GelMod is removed by washing the samples three times in distilled water at 55 °C in order to reveal the structure. **Figure 2****a** shows an SEM image of the fabricated scaffold. In accordance to the used CAD model, an array of 10 by 10 through pores with a square cross-section of 250 µm × 250 µm at a spacing of 300 µm is visible on the large facet of the produced scaffold. From every side of the scaffold, three layers of through pores are visible. An in-depth analysis of the scaffold indicated that the pores are occupied with a fibrous mesh composed of fibers with different diameters (**Figure 2****b, c**). Unlike the scaffold’s struts, the above-mentioned mesh was only visible using SEM and not with optical microscopy. The latter indicates that the mesh volume was too negligible, since it is unable to induce observable changes of refractive index. Since the scaffold pores were not irradiated, we anticipate that the observed mesh is a result of diffusion-driven polymerization (*i.e*., the radicals produced by laser irradiation diffuse and cause GelMod cross-linking outside the exposed region). A recent report by Moroni e*t al.* described the repetitive combination of electrospinning and 3D fiber deposition modeling in order to produce mechanically stable scaffolds with fiber-filled pores [28]. Electrospun nanofibers within the channels provided topographical cues at a nanoscale level and functioned as a sieve facilitating cell entrapment during the seeding procedure. The results indicated that the performance of these scaffolds is superior over scaffolds developed using 3D fiber deposition. Interestingly, one-step scaffold fabrication using 2PP starting from GelMod results in a similar structure.

### 2.3. Primary Human ASC Culture and Differentiation within the Developed Scaffolds

**Figure 3:**
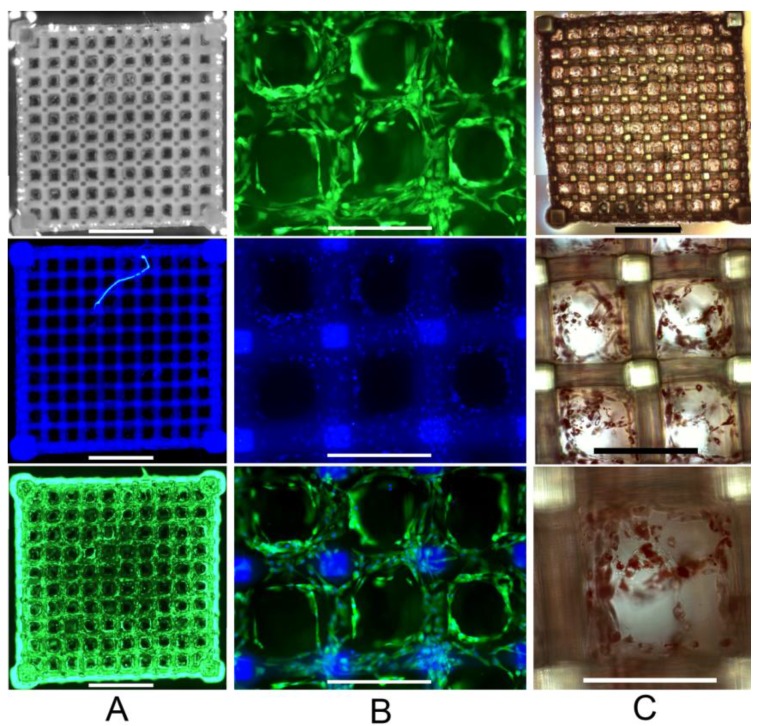
Human adipose-derived stem cells (ASCs) within the 2PP Scaffold at day 7; (**a**) Staining with Calcein AM (*i.e*., cell viability stain, green fluorescence) and Hoechst 33342 (*i.e*., cell nuclei stain, blue fluorescence) indicates the cell distribution (scale bars represent 1 mm); (**b**) Magnified scaffold view (scale bars represent 300 µm); (**c**) Oil Red O staining (*i.e*., indicator of intracellular lipid accumulation) (scale bars represent from top to bottom 1 mm, 300 µm and 200 µm respectively).

Primary human ASCs were drop-seeded onto the scaffold and, after initial cell attachment, cultured in static conditions. After three days in culture, adipogenic medium was added. The cell-seeded scaffolds were stained and analyzed after seven and 22 days. The emission spectrum of Hoechst 33342 cell nuclei stain (*i.e*., blue fluorescence staining) overlaps with the scaffold autofluorescence signal. However, Calcein AM (*i.e*., green fluorescent cell viability staining) reveals a high density of homogeneously distributed vital cells (**Figure 3****a**). At higher magnification, blue stained cell nuclei can be distinguished from the background. The results indicate that the cells are in close proximity to one another. Moreover, they exhibit a spread out morphology and are mainly residing on the scaffolds struts. Fluorescence is observed at different focal planes throughout the scaffold, indicating that the mesh present in the pores does not preclude the cells from migration after the initial seeding procedure. Both Calcein AM stainings as well as the color merge images indicate that fat vacuoles are formed (**Figure 3****b**). Oil Red O staining was used as an intracellular lipid accumulation indicator. The results clearly show the formation of small fat vacuoles within the cells indicating that successful adipose differentiation of ASCs already takes place at day 7 (**Figure 3****c**).

**Figure 4:**
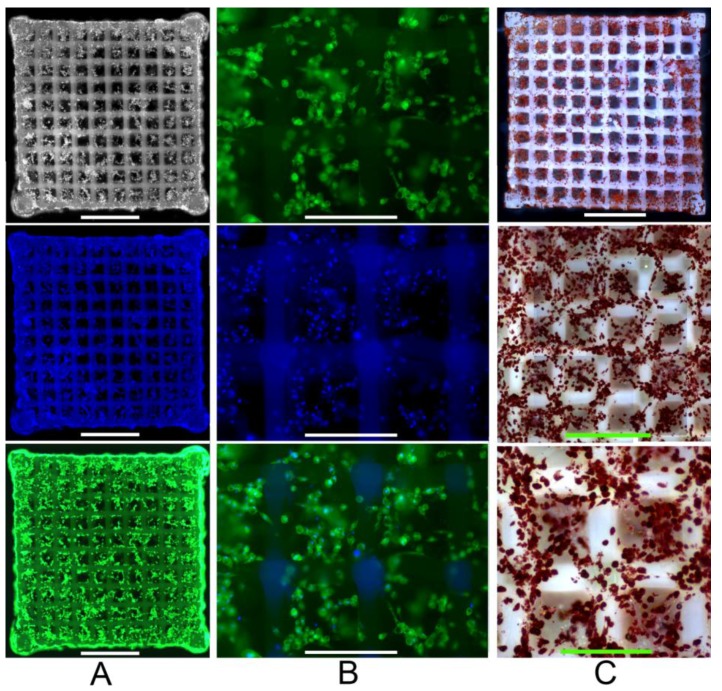
Human ASCs within the 2PP Scaffold at day 22; (**a)** Staining with Calcein AM (cell viability stain, green fluorescence) and Hoechst 33,342 (cell nuclei stain, blue fluorescence) indicates the cell distribution (scale bars represent 1 mm); (**b**) Magnified scaffold view (scale bars represent 300 µm); (**c**) Oil Red O staining (*i.e*., indicator of intracellular lipid accumulation) (scale bars represent from top to bottom 1mm, 600 µm and 300 µm respectively).

The cell number was substantially increased after 22 days (**Figure 4****a**). Most cells were present in clusters accompanied by larger fat vacuoles (**Figure 4****b** and **c**). As a result, both the cell buoyancy as well as the cell cluster formation was increased. The latter aggravates sample handling during the staining procedure, since some cells are lost or relocated. A magnified scaffold section at day 22 shows that in comparison to day 7 many cell groups reside within the scaffold pores. In addition, fat vacuoles are clearly visible in the fluorescence images (**Figure 4****b**). Oil Red staining provides further evidence of an increased neutral fat deposition.

The aim of the present work was to develop precisely defined 3D scaffolds allowing ASC adhesion, proliferation and subsequent differentiation to form adipose tissue. The results indicate that 2PP enables the fabrication of 3D scaffolds starting from GelMod in accordance to the provided CAD model. Gelatin is a proteinaceous material derived from collagen, which is one of the main ECM components of many tissues [29]. As a result, the developed scaffolds mimic the chemical composition of the natural ECM to a great extent. Since preadipocytes are anchorage-dependent cells, the applied material presents a significant advantage. In the present work, we have observed that the 2PP-produced 3D scaffolds starting from GelMod support ASC adhesion and proliferation. Fat deposition indicates the successful ASC differentiation into the adipogenic lineage. Vascularization plays an important role in retaining the functionality of clinically relevant adipose tissue constructs. Masuda *et al*. have already applied gelatin-based injectable microspheres for the sustained release of signaling factors promoting adipose tissue formation *in vivo* [30]. We anticipate that the GelMod-based scaffolds could be applied in a similar way. As a result, the 3D scaffolds could not only act as cell carriers, but also to deliver important signaling factors to be released upon scaffold degradation.

## 3. Experimental Section

### 3.1. Material Synthesis

The detailed description of the synthesis of methacrylamide-modified gelatin (GelMod) can be found elsewhere [25]. In brief, gelatin type B (Bloom strength of 257; Rousselot, Ghent, Belgium) isolated from bovine skin and methacrylic anhydride (Aldrich, Bornem, Belgium) were used as received. In order to obtain a photopolymerizable material, gelatin was chemically modified with methacrylamide side groups. A degree of substitution of 65%, selected for this work, was verified using ^1^H-NMR spectroscopy at 40 °C (data not shown).

A photosensitive polymer solution was prepared by dissolving 1 g GelMod in 5 mL photoinitiator solution at 55 °C. The photoinitiator solution contained 1.5 wt % Irgacure 2959 in DMSO (5% in distilled water).

### 3.2. *In vitro* Degradation Study

The *in vitro* degradation behavior of cross-linked GelMod was investigated by monitoring the weight of the polymerized material in the presence of collagenase (Type I, ≥125 CDU/mg; Sigma-Aldrich, Taufkirchen, Germany) at 37 °C. Freeze-dried samples (1 mm thick and 6 mm in diameter) were first incubated in Tris-HCl buffer (0.1 M, pH 7.4) in the presence of 0.005% (w/v) NaN_3_ and 5 mM CaCl_2_ at 37 °C. After 1 hour, collagenase solution (200 CDU/ml; Tris-HCl buffer) was added to the buffer in a volume relation of 1:1 or 2:1. After 2 hours of initial incubation in collagenase solution, the degradation was stopped at 1 hour intervals by adding EDTA (0.25 M) followed by cooling the samples on ice. Subsequently, the samples were washed three times for 10 min with ice-cooled Tris-HCl buffer and three times with double-distilled water. Finally, the samples were freeze-dried and analyzed gravimetrically.

### 3.3. Scaffold Fabrication Using Two-Photon Polymerization (2PP)

A cavity-dumped oscillator emitting 200 fs pulses at around 515 nm at a repetition rate of 1 MHz was applied. This emission wavelength matches the absorption of the photo-initiator Irgacure 2959 and enables the two-photon absorption process to occur. Laser pulses were focused using a conventional 20x microscope objective (Zeiss, Epiplan NA 0.40). The scaffolds were produced in a layer-by-layer fashion, with the model sliced along the vertical direction at a slice distance of 15 µm. Each layer is produced by a set of parallel laser scans at a distance of 2 µm. The scanning speed was set to 10 mm/s at a constant average laser power of 3.5 mW. Finally, the samples were washed in distilled water at 55 °C to remove unpolymerized material.

### 3.4. Isolation and Culture of Human Adipose-Derived Stem Cells (ASCs)

Human adipose-derived stem cells (ASCs) were isolated from the lipoaspirate of patients undergoing elective plastic surgery after obtaining informed consent. After centrifugation of the lipoaspirate at 920 g for 3 min, the concentrated tissue pieces were washed twice with phosphate-buffered saline (PBS) and digested with 0.1% collagenase type CLS (Biochrom AG, Berlin, Germany) at 37 °C for 45 min. After centrifugation at 450 *g* for 10 min the supernatant was discarded and the sediment was resuspended in PBS. The suspension was filtered through 100 and 40 µm meshes and centrifuged at 350 g for 10 min. Erythrocytes were depleted by applying red blood cell lysis buffer (0.15 M NH_4_Cl, 10 mM KHCO_3_, 0.1 mM ethylene diamine tetraacetic acid (EDTA), pH 7.4). After washing with PBS, the ASC-containing pellet was resuspended in Dulbecco’s modified Eagle's medium/F12 (DMEM/F12, Biochrom AG) supplemented with 10% fetal bovine serum (FBS, Biochrom AG), 100 U/mL of penicillin, 100 µg/mL streptomycin and 10 ng/mL basic fibroblast growth factor (FGF-2, ITC Leibniz University of Hannover, Germany). The isolated cells were seeded into culture flasks and cultivated in culture medium at 37 °C in a humidified atmosphere containing 95% air and 5% CO_2_. ASCs were purified by plastic adherence, non-adherent cells were removed by medium exchange after two days. The medium was changed twice weekly. When the ASCs reached 80% of confluency, they were detached from the culture flasks with 0.05% trypsin/0.02% EDTA for 5 min at 37 °C and subsequently replated for continued passaging. For the cell seeding experiments, ASCs of passage 5–6 were selected.

### 3.5. Scaffold Seeding and ASC Differentiation

ASCs were harvested from the culture flasks with 0.05% trypsin/0.02% EDTA. The cells were pelleted by centrifugation at 450 g for 10 min and resuspended in the culture medium**.** A density of 5 × 10^4^ human ASCs in a small medium volume (30 µL) was seeded onto each sterilized scaffold in separate wells of a 24-well plate and incubated at 37 °C for 1 h to allow initial cell attachment. Afterwards, 1 mL culture medium was added and the cell seeded scaffolds were cultured for 3 days at 37 °C in a humidified atmosphere containing 95% air and 5% CO_2_. Adipogenic differentiation was induced with DMEM supplemented with 10% FBS, 1 µM dexamethasone (Sigma-Aldrich), 10 µg/mL insulin (Sigma-Aldrich), 0.5 mM 3-isobutyl-1-methylxanthine (Sigma-Aldrich) and 0.2 mM indomethacin (Sigma-Aldrich). The medium was changed twice weekly.

### 3.6. Cell Staining and Fluorescence Analysis

Viable cells were stained within the scaffolds using calcein AM (Sigma-Aldrich, green fluorescence staining) and cell nuclei were stained with Hoechst 33342 (Sigma-Aldrich, blue fluorescence staining). The latter was performed by incubating the scaffolds for 15 min at 37 °C with a mixture of 8 μM calcein AM and 10 μg/mL Hoechst 33,342 in culture medium. Afterwards, the scaffolds were washed twice with culture medium followed by fluorescence microscopy analysis using an Axiovert 200 microscope (Carl Zeiss, Oberkochen, Germany) equipped with AxioCam MR and AxioVision Rel. 4.8 software.

### 3.7. Oil Red O Staining for Fat Accumulation

The adipogenic differentiation of ASCs in the cell seeded scaffolds was assessed after 1 and 3 weeks using Oil Red O staining (Sigma-Aldrich) as an indicator of intracellular lipid accumulation. The scaffolds were rinsed in PBS and fixed with 4% neutral buffered formalin (Carl Roth, Karlsruhe, Germany) for 2 hours. After two washing steps with water, the cells were rinsed with 50% ethanol and incubated for 10 min with a filtered saturated solution of Oil Red O in equal parts of acetone and 50% ethanol. Next, the cells were washed in 50% ethanol until only the fat is stained. After two washing steps in water, the cells were analyzed using an Axiovert 200 microscope (Carl Zeiss, Oberkochen, Germany), equipped with AxioCam ICc1 and AxioVision Rel. 4.8 software.

## 4. Conclusions

In the present work, novel 3D scaffolds were developed using 2PP starting from photosensitive gelatin. The results indicate that photopolymerized gelatin preserves its enzymatic degradation capability. In addition, the developed scaffolds are stable in cell culture for over three weeks. Despite the polymeric mesh present in the pores, cell migration throughout the scaffolds remains feasible. In addition to cell adhesion, 2PP scaffolds support human ASC proliferation and differentiation. An indication of ASC differentiation into the adipogenic lineage is provided at day 7 by the observation of intracellular fat vacuoles.
